# The effects of circuit-based resistance training on blood pressure, arterial stiffness, and body composition in community-dwelling older adults: a systematic review and meta-analysis

**DOI:** 10.3389/fphys.2025.1609013

**Published:** 2025-05-20

**Authors:** Zhongxu Hu, Shihua Jiang, Chenhao Hu, Boao Shen, Jinfa Gu

**Affiliations:** ^1^ Moray House School of Education and Sport, University of Edinburgh, Edinburgh, United Kingdom; ^2^ School of Physical Education, Shanghai University of Sport, Shanghai, China; ^3^ School of Physical Education, Henan University, Kaifeng, Henan Province, China; ^4^ China Football College, Beijing Sport University, Beijing, China; ^5^ School of Health Sciences, Universiti Sains Malaysia, Kubang Kerian, Kelantan, Malaysia

**Keywords:** community-dwelling, aging, resistance training, cardiovascular health, body composition

## Abstract

**Background:**

The global aging population has led to a public health crisis, with cardiovascular disease, hypertension, arterial stiffness, and obesity becoming major concerns. Resistance training (RT) has been shown to improve cardiovascular health, but traditional RT has certain limitations.

**Objective and data sources:**

The present systematic review and meta-analysis aims to assess the effects of circuit-based resistance training (CBRT) on blood pressure, arterial stiffness as well as body composition in community-dwelling older adults. PubMed, Cochrane library, Embase, Scopus, and Web of Science, five databases in total, were searched until January 2025. The analysis was restricted to randomized controlled trials.

**Methods:**

A total of 14 studies, involving 704 participants, were included in the meta-analysis. The primary outcomes assessed were arterial stiffness, blood pressure, and body composition.

**Results:**

Significant intervention effects were identified in systolic blood pressure (WMD = −6.10 mmHg, 95% CI: −8.07 to −4.12), diastolic blood pressure (WMD = −2.88 mmHg, 95% CI: −3.96 to −1.81), brachial-ankle pulse wave velocity (WMD = −101.81 cm/s, 95% CI: −136.92 to −66.70), percentage of body fat (WMD = −3.21%, 95% CI: −4.20 to −2.22), fat mass (WMD = −4.83 kg, 95% CI: −8.80 to −0.86), lean body mass (WMD = 1.36 kg, 95% CI: 0.83–1.89), and femoral neck bone mineral content (WMD = 0.27 g, 95% CI: 0.14–0.39). Subgroup analysis by training frequency showed that participants with high training frequency (>3 sessions/week) reduce systolic blood pressure more significantly compared to moderate to low training frequency (≤2 sessions/week) while diastolic blood pressure show no difference between subgroups.

**Conclusion:**

CBRT interventions improve blood pressure, arterial stiffness, and body composition in community-dwelling older adults significantly. Additionally, three sessions of CBRT per week show a superior systolic blood pressure lowering effect.

**Systematic Review Registration:**

PROSPERO, identifier CRD42025646360.

## 1 Introduction

By 2050, the population aged 60 and older worldwide is projected to double, reaching 22% of the global population in the world (Kanasi et al., 2016). The aging process is accompanied by elevated blood pressure and increased arterial stiffness, along with unfavorable changes in body composition (Steen, 1988; Wu et al., 2019). Along with insufficient physical activity, these factors exacerbate chronic health conditions such as hypertension, and cardiovascular disease (Merchant et al., 2021).

Hypertension in the older population is primarily attributed to a decline in arterial wall elasticity, characterized by increased SBP and decreased DBP in older adults (Westhoff et al., 2007). As large arteries progressively stiffen, DBP continues to decline, further elevating SBP and contributing to isolated systolic hypertension (ISH), the most prevalent subtype among the elderly (Asmar, 2003).

Pulse wave velocity (PWV) is widely recognized as the gold standard for assessing arterial stiffness (Laurent et al., 2006). It also serves as an independent indicator of cardiovascular disease in both individuals with existing conditions and in healthy ones (Blacher et al., 1999; Mattace-Raso et al., 2006). Given its strong association with cardiovascular events, PWV has become a key focus in chronic disease prevention among the elderly.

According to the report, 80%–92% of the elderly have at least one chronic illness, while 50%–77% experience multiple chronic conditions (Chiaranai et al., 2018). Additionally, more than 66% of adults aged 65 and older suffered from hypertension (Chobanian et al., 2003). Furthermore, chronic diseases have a significant impact on life expectancy. Eliminating deaths from major cardiovascular diseases could extend life expectancy by approximately 5.5 years (Arias et al., 2013), and alleviate significant burden on the medical system and on relevant departments (Control and Prevention, 2003). Moreover, after the age of 30 in women and 40 in men, bone mineral density begins to decline and continues to decrease throughout life (Santos et al., 2017). With aging, the progressive loss of bone mass and reduced bone strength make the skeleton increasingly susceptible to osteoporosis, thereby raising the risk of fragility fractures (Demontiero et al., 2012). The BMD of femoral neck measured by DXA is a strong predictive indicator of hip fractures (Johnell et al., 2005), which are associated with more than a threefold increase in 1-year motality risk (Klop et al., 2017). Bone mineral content (BMC) refers to the total mass of mineral content within the bone as measured by DXA, expressed in grams (g). BMD, on the other hand, indicates the mineral density per unit area, with the widely used unit being g/cm^2^. From a clinical perspective, however, T-scores and Z-scores, which are derived from raw BMD values, are more commonly used. The T-score is primarily applied to older individuals and represents the number of SDs a person’s BMD deviates from the mean BMD of a healthy young adult population. Defined by the WHO, the T-score is considered the gold standard for diagnosing osteoporosis: a T-score ≥ −1 SD is considered normal, between −1 and −2.5 SDs indicates osteopenia, and ≤−2.5 SDs indicates osteoporosis. The Z-score, in contrast, reflects how much a person’s BMD deviates from the average BMD of individuals of the same age and sex, and is used to evaluate whether a person’s BMD is lower than expected for their age group (Dimai, 2017).

Substantial evidence suggests that exercise, as non-pharmacological interventions, can effectively benefit older adults on their cardiovascular fitness and body composition (Blair, 1996; Ryan et al., 1998; Chodzko-Zajko et al., 2009), enabling them to maintain independence, actively participate in their families and communities, and sustain overall health (Koopman and Van Loon, 2009). The chronic effect of traditional RT on blood pressure management has been widely recognized. However, traditional RT generally show limited improvement on arterial stiffness (Miyachi, 2013; Ashor et al., 2014; Ceciliato et al., 2020; Lopes et al., 2021). Therefore, comprehensive exercise interventions need to be further explored. CBRT is a time-efficient training model that integrates elements of both resistance and aerobic training (Gotshalk et al., 2004; Kolahdouzi et al., 2019). Consequently, CBRT offers various benefits, including enhanced cardiovascular fitness, decreased body fat, improved lean body mass (Gotshalk et al., 2004; Brentano et al., 2008; Camargo et al., 2008; Kolahdouzi et al., 2019). These lower training intensity can minimize muscle soreness and reduce rate of perceived exertion, thereby enhancing compliance and safety among aged individuals (Sparling et al., 1990; Waller et al., 2011).

Community-dwelling older adults, who maintain greater physical independence than hospitalized counterparts, can be well-suited for exercise interventions. However, previous meta-analyses overlooked the effects of CBRT on blood pressure, arterial stiffness. To address this research gap, our systematic review with meta-analysis will evaluate the intervention effects of CBRT on those overlooked outcomes. Additionally, we will evaluate the effects of CBRT on key health indicators across participants with different BMI ranges, as BMI is the most widely used anthropometric measure in clinical practice and research, associating with cardiovascular disease-related mortality (Nuttall, 2015). By systematically evaluating the effects of CBRT on cardiovascular health and body composition in community-dwelling older adults, this study aims to fill the gap in existing knowledge with respect to the effects of this specific form of resistance training in older populations.

## 2 Methods

### 2.1 Protocol and guidance

Following Preferred Reporting Items for Systematic Reviews and Meta Analysis (PRISMA) (Liberati et al., 2009), this study has been registered with PROSPERO (CRD42025646360) to ensure transparency and rigor.

### 2.2 Inclusion criteria

The inclusion criteria were established following the PICOS principles (Liberati et al., 2009). A study was considered eligible if it met the following criteria: all recruited participants were physically independent older adults aged 60 years or older and were community-dwelling rather than hospitalized or institutionalized; CBRT was the only intervention; the control group consisted of individuals who had no exercise intervention; outcome variables included measures related to blood pressure, body composition, and functional autonomy; and only RCTs were included.

### 2.3 Exclusion criteria

Studies were excluded if they met any of the following conditions: Animal studies were used; the study was an abstract, review, or conference article; the study was published in a non-English language; the study contained incomplete data reporting; the study applied other interventions concurrently with CBRT.

### 2.4 Outcomes

The primary outcomes included systolic blood pressure (SBP), diastolic blood pressure (DBP), brachial-ankle pulse wave velocity (baPWV), percentage of body fat (%BF), fat mass (FM), lean body mass (LBM), body mass index (BMI), femoral neck bone mineral density and content (BMD_FN_ and BMC_FN_).

### 2.5 Search strategy

In order to examine the effects of CBRT on cardiovascular and body composition in community-dwelling adults, One of the authors (JSH) conducted the search of several databases: PubMed, Cochrane library, Embase, Scopus, and Web of Science, from the inception of databases to 10 January 2025. The search strategy consists of both specific MESH terms and entry terms “Circuit-based,” “Training” or “Exercise,” and “older adults,” which are combined with Boolean logic terms “AND,” “OR,” “NOT.”

### 2.6 Data collection process

After removing duplicates, two independent reviewers (SBA and HCH) screened titles and abstracts to exclude ineligible studies. If an abstract lacked sufficient information to determine eligibility, the full text was retrieved and assessed. Data from all included studies were then extracted using a standardized data extraction form. Any disagreements were resolved through consensus.

### 2.7 Assessment of risk of bias and publication bias

Two researchers independently assessed the quality of all included trials using the Cochrane Collaboration’s Risk of Bias Tool (Higgins and Altman, 2008). The quality of the included articles was assessed using RevMan 5.4, which evaluates seven domains: random sequence generation, allocation concealment, participant blinding, assessor blinding, completeness of outcome data, selective reporting, and other potential biases.

Due to the limited number of studies per outcome (maximum of six studies), publication bias was not assessed using funnel plots or Egger’s test, as these methods require a minimum of 10 studies for reliable interpretation, according to Cochrane recommendations (Liberati et al., 2009). Any discrepancies were discussed among the three reviewers until a consensus was reached.

### 2.8 Data synthesis

All the outcomes included in this meta-analysis were continuous, and we standardized all the outcomes into means ± SD if they were demonstrated as means ± SE, or means with 95% CI (Altman and Bland, 2005). For each study, effect sizes were calculated for both the CBRT and control groups. A correlation coefficient of 0.50 was used, and effect sizes were computed using the following formula (Higgins et al., 2008):

$${\text{SD}}_{\text{change}}=\sqrt{SD_{\text{baseline}}^{2}+SD_{\text{post}}^{2}-\left(2\times \text{Corr}\times {\text{SD}}_{\text{baseline}}\times {\text{SD}}_{\text{post}}\right)}$$

We conducted statistical analyses using R (version 4.4.3). *I* ^2^ test was used to assess heterogeneity, where *I* ^2^ values indicated the degree of heterogeneity (Higgins and Thompson, 2002). If any degree of heterogeneity was detected (*I* ^2^ > 0%) and subgroup analyses failed to adequately explain the heterogeneity, both fixed-effect and random-effects models were applied. The results from both models were demonstrated in forest plots to ensure transparency. However, in line with a conservative approach, the random-effects model was adopted for reporting the main findings to account for potential heterogeneity across studies. This approach enhances the robustness and transparency of the meta-analysis by considering the model-based uncertainty.

### 2.9 Subgroup analysis

In order to further investigate the BP-lowering effect of CBRT with different training frequency, the studies were categorized into two subgroups: high training frequency (3 sessions/week) and moderate to low training frequency (≤2 sessions/week). The effect of the CBRT intervention was then analyzed separately for each subgroup.

### 2.10 Sensitivity analysis

To assess the robustness of our findings, we conducted a series of sensitivity analyses.

Leave-one-out analysis: Each study was systematically removed one at a time to determine its influence on the pooled effect size and heterogeneity; Comparison of fixed-effects and random-effects models: To evaluate the consistency of our findings across statistical models, we compared the results obtained using fixed-effects and random-effects models. If any of these analyses resulted in substantial changes in the pooled effect size or statistical significance, these findings were documented and discussed.

## 3 Results

### 3.1 Eligible studies and study characteristics

**FIGURE 1:**
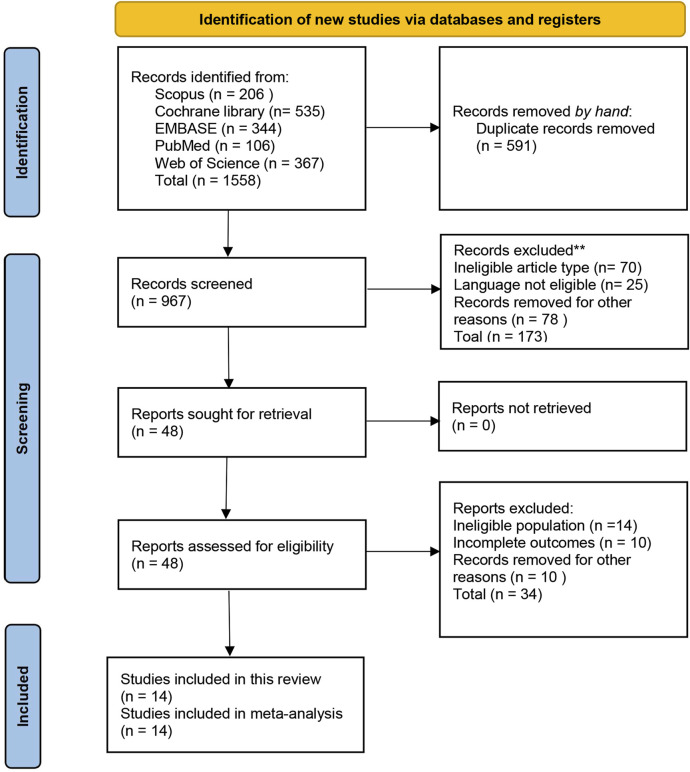
PRISMA flow diagram of study selection process.

The initial search identified 1,558 articles, with 591 duplicates were removed. The titles and abstracts of the remaining 967 articles were screened, leading to the exclusion of 919 articles. A full-text review for the 48 articles remained was conducted, identifying 14 studies that satisfy the inclusion criteria for this systematic review (Rhodes et al., 2000; Miura et al., 2008; Bocalini et al., 2012; Romero-Arenas et al., 2013; Miura et al., 2015; Venturelli et al., 2015; Roberson et al., 2018; Jung et al., 2019; Marcos-Pardo et al., 2019; Choi et al., 2020; Camacho-Cardenosa et al., 2022; Jung et al., 2022; Al-Mhanna et al., 2024; Simms et al., 2024). The meta-analysis was then conducted using outcome measures from these included studies (Figure 1).

Of the 14 included articles, a total of 704 participants aged 60–75 years were involved. There were 19 CBRT intervention groups (390 individuals) and 17 control groups (314 individuals). Table 1 summarizes the main characteristics of all included studies.

**TABLE 1:** Main characteristics of included studies in the meta-analysis.

Study	Group (n)	F%	Age	BMI	Summary of intervention details			
					Intervention	Duration	Exercises/sets/reps	Outcomes
Al-Mhanna et al. (2024)	TG(34)	55.7%	62.60 ± 6.90	32.41 ± 4.30	12/3/36	20–60	7/2-4/15-30	BMI
	CG(35)		61.70 ± 5.20	32.81 ± 5.60	-	-		
Bocalini et al. (2012)	TG_AW(18)	100%	66 ± 4	18.5–24.9	12/3/36	50	12/NR/NR	%BF, FM, LBM
	TG_OW(14)		64 ± 4	25.0–29.9	12/3/36	50		
	TG_O(9)		62 ± 2	>30.0	12/3/36	50		
	CG_AW(9)		67 ± 9	18.5–24.9	-	-		
	CG_OW(10)		63 ± 2	25.0–29.9	-	-		
	CG_O(9)		62 ± 1	>30.0	-	-		
Camacho-Cardenosa et al. (2022)	TG_N(17)	59%	70.35 ± 3.37	24.82 ± 2.45	24/3/72	45	9/3/12-15	BMD_FN_, BMC_FN_
	TG_H(13)		68.46 ± 3.82	26.41 ± 3.46	24/3/72	45		
	CG(20)		70.55 ± 4.10	28.23 ± 3.06	-	-		
Choi et al. (2020)	TG(15)	NR	75.1 ± 1.4	25.3 ± 0.5	12/3/36	60	19/NR/10-12	SBP, DBP, MAP, PP
	CG(12)		72.3 ± 1.4	25.5 ± 0.4	-	-		
Jung et al. (2019)	TG(13)	100%	75.0 ± 3.9	21.8 ± 1.5	12/3/36	25–75	PRO 10/1-3/NR	%BF, FM, LBM, BMI
	CG(13)		74.9 ± 5.2	21.9 ± 1.5	-	-		
Jung et al. (2022)	TG(13)	100%	75.36 ± 4.50	22.50 ± 1.75	12/3/36	45–75	PRO 10/1-3/NR	SBP, DBP, MAP, PP, baPWV, %BF, LBM, BMI
	CG(13)		74.64 ± 5.77	22.58 ± 1.69	-	-		
Marcos-Pardo et al. (2019)	TG(24)	60%	M: 69 ± 3.2	27.97 ± 3.74	12/3/36	Not fixed	6/NR/8-12	%BF, LBM, BMI, FA
			F: 70 ± 4.1	26.67 ± 4.51	-	-		
	CG(21)		M: 69 ± 3.2	27.57	12/3/36	Not fixed		
			F: 70 ± 4.1	26.09 ± 3.34	-	-		
Miura et al. (2008)	TG_1DW(29)	100%	69.0 ± 6.5	22.8 ± 2.4	12/1/12	40	8/3/8	SBP, DBP, baPWV, BMI
	TG_2DW(25)		69.5 ± 7.0	23.5 ± 2.7	12/2/24	40		
	CG(23)		68.9 ± 7.5	23.7 ± 3.0	-	-		
Miura et al. (2015)	TG_HTN(45)	100%	72.0 ± 7.1	NR	12/2/24	90	PRO 6-8/3-5/15-20	SBP, DBP, baPWV, %BF
	CG_HTN(47)		71.8 ± 5.6	NR	-	-		
	TG_NT(55)		72.9 ± 5.7	NR	12/2/24	90		
	CG_NT(53)		69.7 ± 6.7	NR	-	-		
Rhodes et al. (2000)	TG(20)	100%	68.8 ± 3.2	NR	52/3/156	60	8/3/8	BMD_FN_, BMC_FN_
	CG(18)		68.2 ± 3.5	NR	-	-		
Roberson et al. (2018)	TG(9)	73.3%	72 ± 3	33.0 ± 1.0	12/3/36	Not fixed	11/NR/12	SBP, DBP
	CG(7)		70 ± 3	31.1 ± 2.4	-	-		
Romero-Arenas et al. (2013)	TG(16)	NR	62.1 ± 6.3	29.7 ± 4.1	12/2/24	35–47	PRO 6/1-3/6-12	BMD_FN_
	CG(7)		58.0 ± 5.0	29.9 ± 5.8	-	-		
Simms et al. (2024)	TG(11)	55.5%	70.3 ± 5.7	NR	8/3/24	40–45	PRO 10/1-3/10-12	SBP, DBP, MAP
	CG(7)		71.6 ± 5.2	NR	-	-		
Venturelli et al. (2015)	TG(10)	50%	67 ± 4	27 ± 3	12/3/36	60	4/NR/NR	BMI
	CG(10)		66 ± 7	27 ± 6	-	-		

“-” stands for not receiving intervention, TG, training group; CG, control group; NR, not reported; PRO, progressive training; AW, appropriate weight; OW, overweight; O, obese; 1DW, 1 day per week; 2DW, 2 days per week; N, normoxic training condition; H, hypoxic training condition; HTN, hypertensive population; NT, normotensive population; M, male; F, female.

Due to data limitations, one study (Romero-Arenas et al., 2013) reported that the mean age of the control group was 58 years, while that of the experimental group was 62.1 years. Since their mean age met the inclusion criteria (≥60 years), we still considered the study eligible. Eight included studies focused exclusively on community-dwelling older female (100% female participants), while the remaining studies adopted a mixed-gender approach, and no study specifically examined older male.

### 3.2 Quality (risk of bias) assessment

**FIGURE 2:**
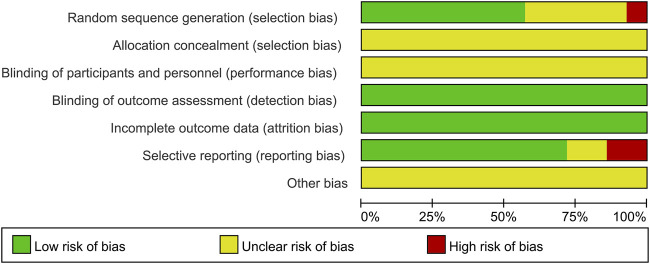
Proportional representation of risk of bias assessment in included trials.

**FIGURE 3:**
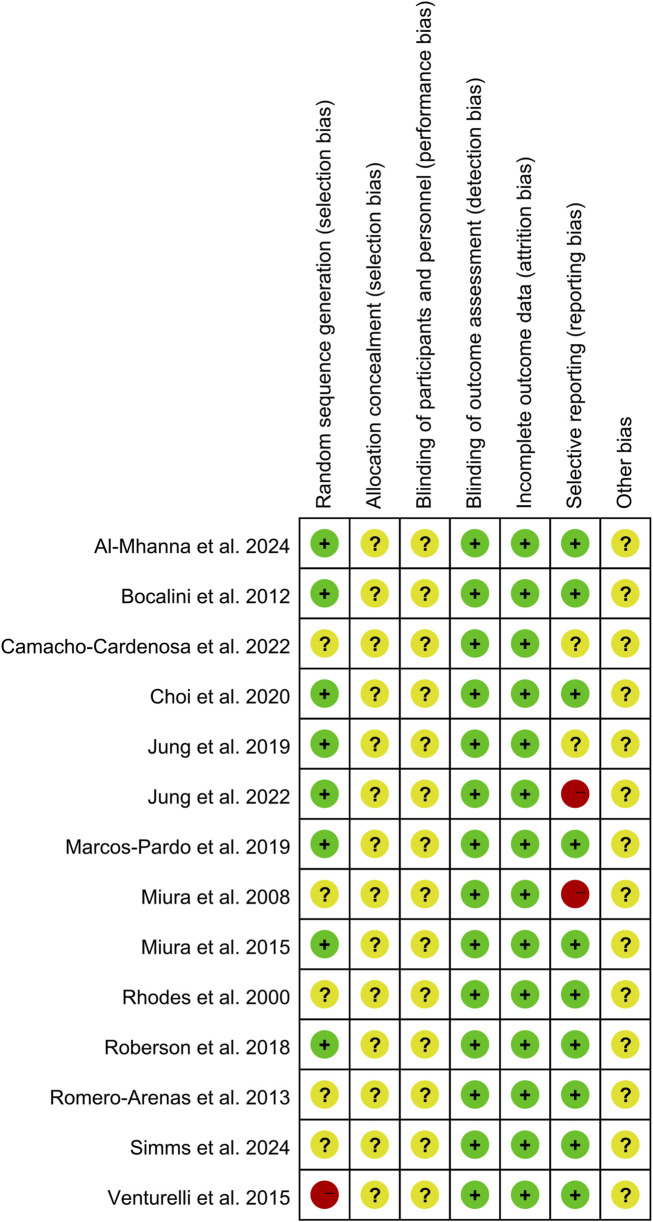
Risk of bias summary: review authors’ judgments about each risk of bias item for each of the 14 included studies.

Detailed risk of bias assessment results are provided (Figures 2, 3). In exercise intervention studies, blinding of participants is generally not feasible; therefore, this domain was assessed as unclear risk. Among the included studies, five did not provide information on the randomization method (unclear risk of bias), while two did not use randomization and were therefore rated as high risk. None of the included studies reported on allocation concealment (unclear risk of bias). Additionally, two studies did not report their pre-registration information, leading to a high risk rating for selective reporting.

### 3.3 Results of the meta-analysis

#### 3.3.1 Effect of circuit-based resistance training on blood pressure

**FIGURE 4:**
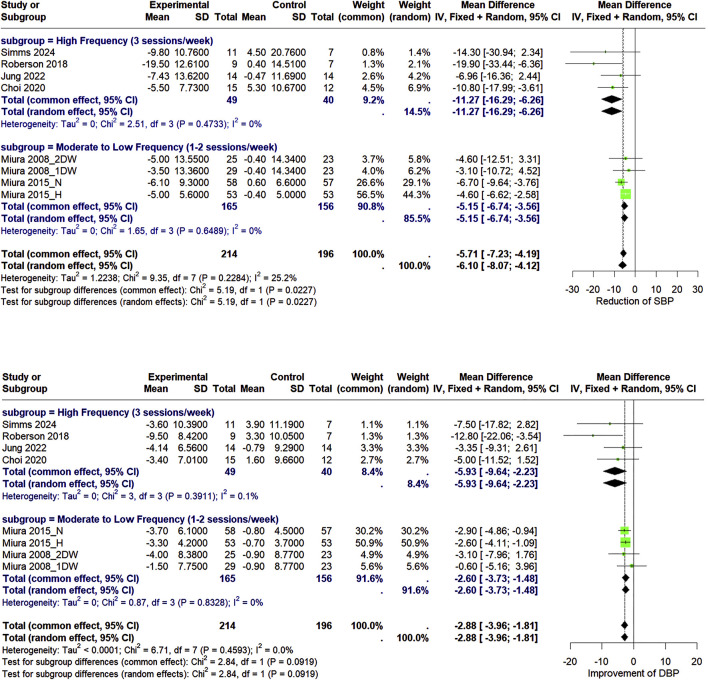
Forest plot of the pooled effects of CBRT versus control on SBP, DBP in older adults living in the community using both fixed-effects and random-effects model. “1DW” and “2DW” indicate training frequencies of 1 or 2 days per week, while “H” and “N” represent hypertensive and normotensive populations.

After conducting the analysis of data from all included studies, we found that the CBRT group showed a significant reduction in SBP (WMD = −6.10 mmHg, 95% CI: −8.07 to −4.12, *I* ^2^ = 25.2%), DBP (WMD = −2.88 mmHg, 95% CI: −3.96 to −1.81, *I* ^2^ = 0%) compared to the control group (Figure 4).

#### 3.3.2 Effect of circuit-based resistance training on arterial stiffness

**FIGURE 5:**
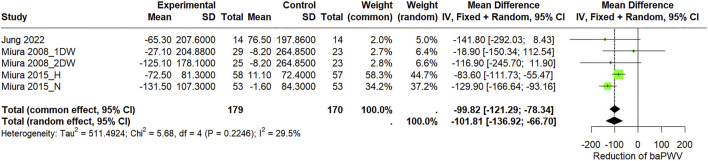
Forest plot of the pooled effects of CBRT versus control on baPWV in community-dwelling older individuals using both fixed-effects and random-effects model. “1DW” and “2DW” refer to training frequencies (1 or 2 days per week), while “H” and “N” indicate hypertensive and normotensive populations.

The meta-analysis (Figure 5) of three included studies of baPWV revealed that CBRT reduces baPWV (WMD = −101.81 cm/s, 95% CI: −136.92 to −66.70, *I* ^2^ = 29.5%).

#### 3.3.3 Effect of circuit-based resistance training on body composition

**FIGURE 6:**
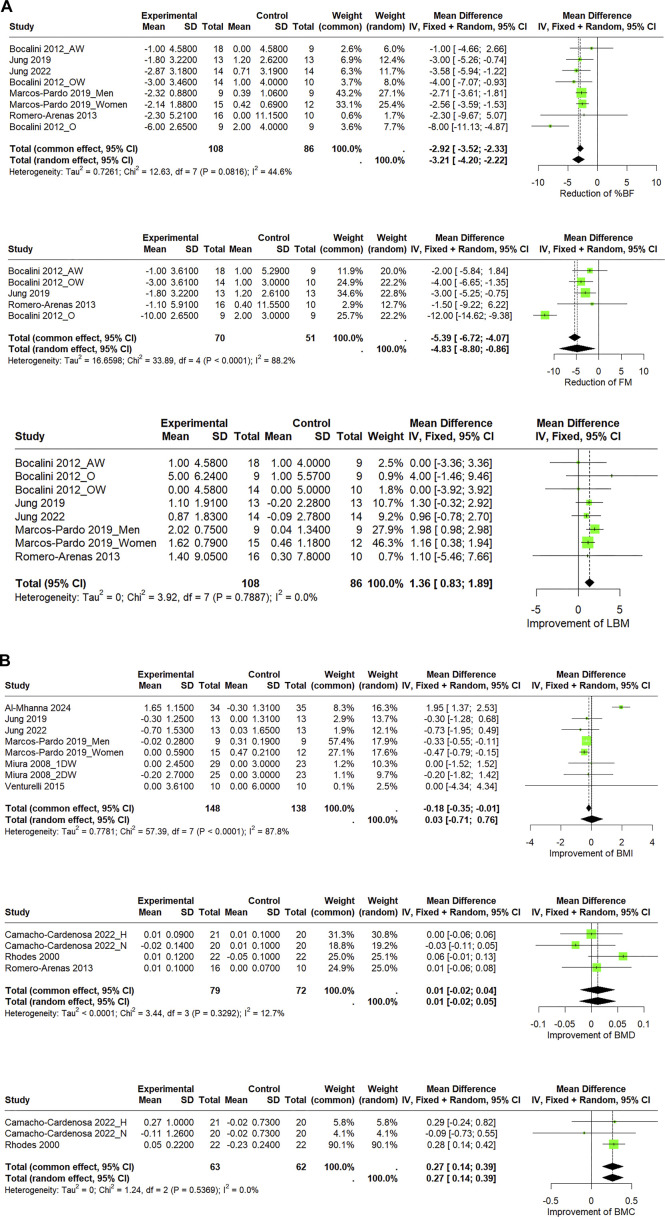
**(A)** Forest plot of the pooled effects of CBRT versus control on body composition in community-dwelling older adults using both fixed-effects and random-effects model. “AW,” “OW,” and “O” denote BMI categories. **(B)** Forest plot of the pooled effects of CBRT versus control on body composition in community-dwelling older adults using both fixed-effects and random-effects model. “1DW” and “2DW” indicate training frequencies; “H” and “N” represent interventions under hypoxic and normoxic conditions.

The meta-analysis revealed a significant overall effect of CBRT in decreasing %BF (WMD = −3.21%, 95% CI: −4.20 to −2.22, *I* ^2^ = 44.6%), FM (WMD = −4.83 kg, 95% CI: −8.80 to −0.86, *I* ^2^ = 88.2%), BMI (WMD = 0.03 kg/m^2^, 95% CI: −0.71 to −0.76, *I* ^2^ = 0%), and increasing LBM (WMD = 1.36 kg, 95% CI: 0.83–1.89, *I* ^2^ = 0%), BMD_FN_ (WMD = 0.01 g/cm^2^, 95% CI: −0.02 to 0.05, *I* ^2^ = 12.7%), BMC_FN_ (WMD = 0.27 g, 95% CI: 0.14–0.39, *I* ^2^ = 0%) (Figure 6).

### 3.4 Sensitivity analyses

We conducted leave-one-out sensitivity analyses for outcomes of BMI (Supplementary Figure S1), %BF (Supplementary Figure S2), and FM (Supplementary Figure S3), to explore potential sources of heterogeneity. The analyses demonstrated that the exclusion of this study significantly reduced heterogeneity (from *I* ^2^ = 87.8%–0%) and changed the overall effect estimate from a non-significant mean difference of 0.03 (95% CI: −0.71 to 0.76) to a significant reduction of −0.37 (95% CI: −0.55 to −0.20). Given the determinant influence of this single study, the conclusion should be interpreted with caution.

Leave-one-out sensitivity analysis for %BF and FM identified the experimental arm “Obese group” from Bocalini et al. (2012) as a potential contributor to the observed moderate-to-high heterogeneity (*I* ^2^ = 44.6% and 88.2%). However, the exclusion of this study or any other single study from both outcomes, did not substantially alter the overall effect size or change the statistical significance of the pooled results. Therefore, all studies were retained in the main analysis. Given the presence of moderate heterogeneity, a random-effects model was adopted to provide a more conservative and robust estimate of the effect size.

### 3.5 Subgroup analysis

In order to further investigate the BP-lowering effect of CBRT with different training frequency, the studies were categorized into two subgroups: high training frequency (3 sessions/week) and moderate to low training frequency (≤2 sessions/week). Subgroup analyses demonstrated that high-frequency CBRT was significantly more effective in reducing systolic blood pressure (SBP) compared to moderate-to-low frequency CBRT (*p* for subgroup differences <0.05). Although high-frequency CBRT also showed greater reductions in diastolic blood pressure (DBP), the difference between the two subgroups was not statistically significant (*p* = 0.09).

## 4 Discussion

To the best of our knowledge, this is the first meta-analysis systematically evaluating the effects of CBRT on blood pressure, arterial stiffness, and bone health in community-dwelling older adults.

Our findings suggest that CBRT can be safely applied to improve cardiovascular health and body composition in older populations. Notably, seven studies included participants aged 70 years or more, yet still demonstrated beneficial effects. Among the 14 studies included in this meta-analysis, 11 focused on the effects of exercise interventions on cardiovascular health and body composition. The intervention period for these studies was uniformly 12 weeks, except for Simms et al. (2024), which lasted 8 weeks. In contrast, studies targeting bone health typically required longer intervention durations of 12, 24, or 52 weeks (Rhodes et al., 2000; Romero-Arenas et al., 2013; Camacho-Cardenosa et al., 2022). Therefore, discussions regarding the improvements in cardiovascular function are based solely on short-to medium-term interventions. CBRT, as an “optimal combination” of training modalities, led to a significant reduction in SBP (−6.10 mmHg), DBP (−2.88 mmHg), and baPWV (−101.81 cm s^−1^). This may be due to the nature of CBRT, where lighter weights are lifted with shorter recovery time between sets and result in greater cardiovascular stimulation (Gotshalk et al., 2004).

Our meta-analysis demonstrated that CBRT significantly improves both blood pressure and arterial stiffness at the same time. In consistent with ours, a meta-analysis reported that low-intensity short-rest resistance training significantly reduces baPWV by improving endothelial function (Okamoto et al., 2011). Moreover, resistance training, regardless of intensity, has been shown to effectively improve blood pressure, including both peripheral and central blood pressure (Figueroa et al., 2019). In contrast, traditional resistance training with heavy weights has no significant effect on arterial stiffness in middle-aged and older adults (Miyachi, 2013; Ashor et al., 2014; Lopes et al., 2021). One potential mechanism potentially interpreting the effect of CBRT is that the CBRT-induced muscle hypoxia stimulates nitric oxide (NO) production, enhances endothelial function, and subsequently reduces arterial stiffness, whereas high-intensity resistance training with longer recovery periods does not improve this function (Okamoto et al., 2009). It is suggested that lower-intensity resistance training with shorter rest intervals may be more effective in simultaneously improving baPWV and blood pressure. In terms of training organization, CBRT inherently possesses these characteristics.

It is reported that 10 mmHg reduction in SBP or SBP decrease to lower than 130 mmHg can greatly lower the risk of major cardiovascular disease and coronary heart disease (Ettehad et al., 2016). Additionally, a meta-analysis stated that every 1 m/s increase in baPWV can compound the risk of developing cardiovascular increases by 12% (Vlachopoulos et al., 2012). In this meta-analysis, CBRT significantly lower the baPWV by around 1 m/s, which can be interpreted as a significant lowered risk of cardiovascular disease. However, Since the included studies in this meta-analysis reported blood pressure-related outcomes only lasted 8–12 weeks, the long-term sustainable effect of CBRT-induced cardiovascular fitness improvements in community-dwelling older adults remains unclear. Furthermore, the significant reduction in baPWV observed in this study were restricted to suggest that CBRT may improve peripheral arterial elasticity, while its effects on central aortic compliance remain to be elucidated. Future studies should incorporate carotid-femoral pulse wave velocity (cfPWV) assessments to further investigate the effect of CBRT on lowering central arterial stiffness.

Body composition primarily includes fat, muscle, bone, and water. These components together constitute the total body mass. The relative proportion of body composition can reflect the physiological status and overall health of an individual (Heymsfield et al., 2005). The present meta-analysis indicates that CBRT has positive effects in reducing body fat and improving LBM. This effect may be attributed to the CBRT model, which enhances glucose uptake by skeletal muscle, reduces fat storage in adipose tissue, and helps maintain or increase basal metabolic rate (Donnelly et al., 2009).

Although the meta-analysis revealed a significant improvement in BMC_FN_, no significant change was observed in BMD_FN_. The increase in BMC_FN_ suggests a potential enhancement in bone quality at the femoral neck; however, due to the absence of clinically diagnostic indicators such as T-scores, this finding can only be interpreted as evidence of the physiological potential of CBRT to improve bone mineral content. Its clinical relevance in terms of osteoporosis prevention or treatment remains unclear. This is in line with previous findings suggesting that low-impact resistance training may not produce significant changes in BMD_FN_ or BMD at lumber spine in older adults (Marques et al., 2012). In contrast, a 6-month high-intensity resistance training intervention has shown that BMD_FN_ could be improved significantly (Vincent and Braith, 2002). This may be attributed to the relatively light weights used in CBRT, which may not impose the mechanical stress to stimulate BMD increases, thereby limiting the potential for BMD improvement.

## 5 Limitation

Although this study provides important evidence. Some limitations in the present meta-analysis also need to be acknowledged.

Firstly, the included studies showed overall low quality with quite a few unclear or unreported risk of bias, which may have compromised the robustness of our findings and limited the generalizability of the results. Secondly, only a few studies reported on several outcomes variables, such as baPWV, which restricted our ability to conduct more comprehensive subgroup analyses. Another potential limitation of this meta-analysis is the inability to assess publication bias due to the small number of included studies. Future studies with sufficient number of included study could confirm the robustness of these findings and further evaluate possible publication bias.

## 6 Conclusion

This meta-analysis suggests that CBRT is a safe and effective intervention in community-dwelling older adults, which can significantly improve elevated blood pressure, stiffened arteries, and exacerbated body composition with age. An 8–12-week CBRT program can effectively improve systolic and diastolic blood pressure, as well as body fat percentage and lean body mass in community-dwelling older adults. A higher training frequency (three sessions per week) appears to exert a more pronounced effect on blood pressure management in this population. Exercise prescriptions based on the modality of CBRT can be designed for community-dwelling older adults to promote public health in communities.

## Data Availability

The raw data supporting the conclusions of this article will be made available by the authors, without undue reservation.
