# Supplementary material for: The effects of circuit-based resistance training on blood pressure, arterial stiffness, and body composition in community-dwelling older adults: a systematic review and meta-analysis
Source: Front Physiol. 2025 May 20;16:1609013. doi: 10.3389/fphys.2025.1609013 (PMC12129804; doi:10.3389/fphys.2025.1609013)
Supplement: Supplementary file 1 [file DataSheet1.pdf]

Sensitivity Analysis

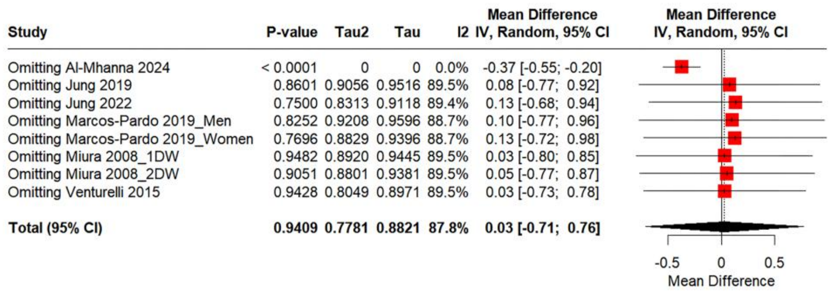

Figure S1: Sensitivity analysis for BMI using a leave-one-out approach. Exclusion of *Al-Mhanna et al. (2024)* markedly reduced heterogeneity and changed the direction and significance of the overall effect.

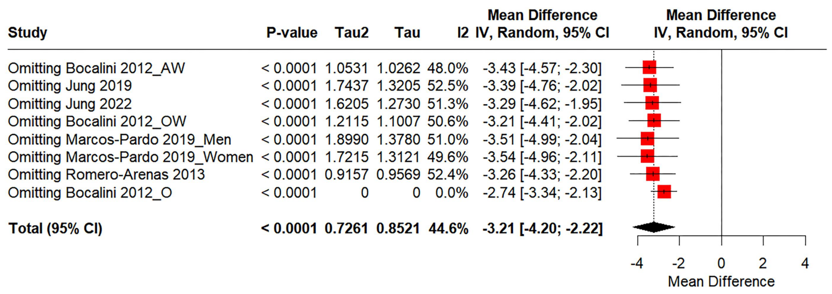

Figure S2: Sensitivity analysis for %BF using a leave-one-out approach. Exclusion of *Bocalini 2012_O* reduced heterogeneity, but by omitting any single one study did change the direction and significance of the overall effect.

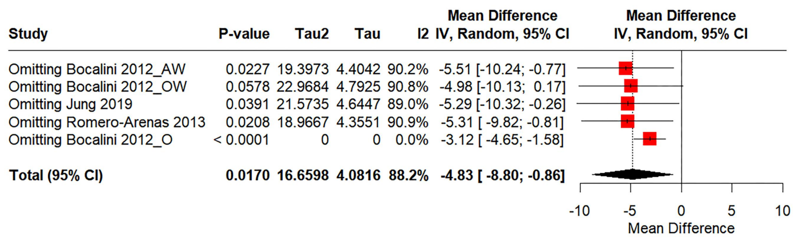

Figure S3: Sensitivity analysis for FM using a leave-one-out approach. Exclusion of *Bocalini 2012_O*

reduced heterogeneity, but by omitting any single one study did change the direction and significance of the overall effect.
